# Splitting of droplet with different sizes inside a symmetric T-junction microchannel using an electric field

**DOI:** 10.1038/s41598-022-07130-6

**Published:** 2022-02-25

**Authors:** Keivan Fallah, Ehsan Fattahi

**Affiliations:** 1 Department of Mechanical Engineering, Sari Branch, Islamic Azad University, Sari, Iran; 2 Brewing and beverage technology, TUM School of Life Sciences, Technical University of Munich, Freising, Germany

**Keywords:** Nanoscience and technology, Engineering

## Abstract

In the current study, droplets dynamics under an asymmetric electric field in a T-junction are numerically studied using COMSOL Multi-physics software. The effect of different factors such as dimensionless length of mother droplet (L^*^), Capillary number (Ca), and electric capillary number (Ca_e_) are investigated on the breakup process in symmetric T-junctions. Two novel patterns of droplets, namely, hybrid asymmetric splitting mode and sorting patterns, have been observed by imposing an electric field in one branch of the microchannel. It is also concluded that using an electric field is a promising strategy to reach droplets with arbitrary sizes and control over the splitting ratio of daughter droplets precisely in a T- junction by adjusting the electric field strength. After a certain electric capillary number ($\left. {Ca_{e} } \right|_{Sorting}$), the mother droplet does not breakup and is sorted on the side of the branch that the electric field imposes. Furthermore, $\left. {Ca_{e} } \right|_{Sorting}$ increases with the increment of L^*^ and Ca.

## Introduction

Nano and micro droplet formation has attracted attention in recent years due to its vast application in industries such as drug delivery^1^, pharmaceutical ^2^, and food industry^3^. The ability to control the droplet size and sort them introduces many advantages such as cost reduction, higher efficiency, and improved safety in such systems. Aspects of droplet behavior such as coalescence^4^, trapping^5^, deformation^6^, fission^7,8^, formation^9–18^, and breakup^19–31^ were explored by researches in several geometries in a microfluidic network involving flow-focusing^9,14,17^, cross junction^12^, T-junction^10,11,18^, Co-flowing^7,13^, etc. The main approach to tune the size of droplets are T- and Y-junctions^19–31^.

The motion of the droplets can be modulated passively or actively. In passive microdevices, the droplet size significantly relies on the channel junctions and working fluids^19–24,26,28^. Link et al.^19^ introduced two strategies to split up droplets asymmetrically. They used an isolated inside a straight channel. The primary disadvantage of this strategy is that a different process is needed to separate the generated small and large droplets after producing in which move together along the channel. Also, they proposed another available strategy for droplet breakup with different sizes by using a T-junction with different arms. Three different regimes were observed as a function of the droplet length and capillary number, including no breakup, breakup with tunnels, and breakup without tunnels obstruction^22^. It must be considered that both the pressure droplet and the manufacturing cost are increased in this system because the length of the arms must be increased. Ménétrier-Deremble and Tabeling^20^ experimentally investigated the asymmetric breakup of droplets in a λ-junction microchannel with arbitrary angles. They reported that the breakup volume ratio depends on the flow geometry only and is independent of the fluid characteristics and the flow conditions. The drawback of this method is that this method cannot produce the low volume ratio of droplets. Leshansky and Pismen^23^ presented a correlation for the critical droplet length (*l*_*Cr*_) as a function of Ca number as $\frac{{l_{Cr} }}{w} = 1.3 Ca^{ - 0.21} { }$. This relation showed a very good agreement with the numerical and experimental results. Subsequently, this critical threshold was experimentally verified by Jullien et al.^22^. Similar to the correlation of Leshansky and Pismen^23^, Fu et al.^24^ proposed an improved power–law relationship as $\frac{{l_{Cr} }}{w} = a Ca^{b}$. Where *a* and *b* are the fitting parameters which depend on the channel geometry and the viscosity ratio of the fluids. Bedram et al.^26^ numerically investigated the asymmetric breakup of droplets in T-junction microchannel with valve in one of the arms. They found out that smaller droplets in the arm with valve is generated by decreasing the capillary number. Moqadam et al*.*^28^ used a titled slat in the center of micro- and nano-scaled T-junctions to control the breakup ratio of droplets. They reported that their proposed system can generate droplets with small volume ratios, while the available methods are not able to achieve.

Although this method can be fabricated easily, any change in size requires new geometry and configuration. This increases the cost and reduces the flexibility of the system. Also, sorting is an issue (big and small droplets are passing the same way). There is a limit in the size ratio of the originated droplets to provide droplets with the desired size. Hence, the active technique is suggested to regulate droplets. This technique is achieved by applying an additional energy field involving temperature field^21^^,^ magnetic field^13,27^, micro-valves^25,31^, and electric field^14,17,29,30^. Besides the benefit of fast response time, another advantage of this method is that it is easy and reliable to precisely control the droplet's size and flexibility in changing the size. Ting et al.^21^ introduced a novel method for producing unequal-sized droplets by setting a heater at one of the branches of a T-junction microchannel. The disadvantage of this method is that the temperature does not reach values larger than 40˚C. Thus, its application is limited, especially for biological applications. Yoon et al.^25^ reported that mother droplet could be divided into daughter droplets in a wide range of the volume ratio by using two pneumatic valves located downstream of the bifurcating microchannel. This method has the main disadvantage as the structure of the valve affects the flow pressure and leads to difficulties in controlling the droplet breakup with a specified volume ratio. Ma et al.^27^ experimentally studied the breakup dynamics of ferrofluid droplets in T-junction microchannels induced by the magnetic field. They found that the magnetic force could affect the droplet breakup process and classified three different regimes: breakup without obstruction, breakup with part obstruction, and breakup with permanent obstruction. The main disadvantage of this mechanism is that generated droplets are equal, whereas, in some industries, like the pharmaceutical industry, droplets in various sizes are needed. Agnihotri et al.^31^ analyzed the capability for selective breakup at two locations using a T-junction and expansion channel by locally reducing the main channel width. They reported four different patterns: no droplet breakup in both junctions, droplet breakup in the first junction, droplet breakup in both junctions, and droplet breakup in the first junction onset of breakup in the second junction.

Several studies have shown that using an electric field as active control of droplets is more robust and faster than other active control methods. Link et al*.*^29^ were one of the first groups to experimentally explain that applying an electric field is an effective strategy to manipulate and control the breakup, sorting, and coalescence in the microchannel. They applied an electric field parallel to the flow after the intersection of the symmetric T-junction microchannel. Jafari and Fallah^30^ numerically investigated the effects of electric field on droplet breakup in a symmetric T-junction microchannel. They showed that droplet splits faster in the presence of an electric field compared to the case without the electric field at the same condition. Yin et al.^17^ applied an AC electric field to adjust the droplet formation in the flow-focusing device. They found that the electrical frequency is directly correlated with the droplet formation regime's transition. Hatami et al.^14^ numerically modeled the droplet formation in the flow-focusing device under an electric field. Hatami et al.^14^ and Yin et al.^17^ do not work based on the breakup of droplets in which mother droplets split up two daughter droplets. Also, these methods generate droplets of one size, whereas they have the disadvantage of the method suggested by Ma et al.^27^.

This study proposes a new technique to use an electric field in a symmetric T-junction under the electric field in microchannels to regulate the size of the droplet and control the breakup speed, and sort the droplets simultaneously. Moreover, the effect of the electrical field on droplet breakup and the feasibility of applying an electrical field to control the size and breakup speed in droplet formation in microchannels is investigated numerically. The study is conducted by investigation of the important non-dimensional parameters involving electric Capillary number (Ca_e_), droplet length (L^*^), and Capillary number (Ca) in detail.

## Problem description

**Figure 1:**
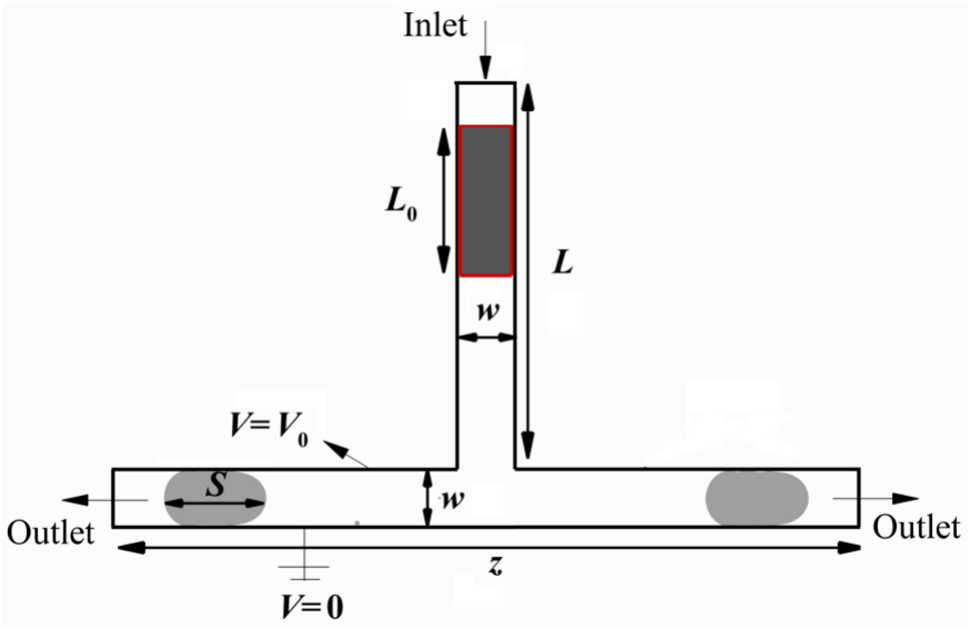
A schematic illustration of 2D symmetric T-junction microchannel.

This study investigates the droplet deformation of a viscous fluid flowing in a symmetric T-junction microchannel (Fig. 1) with non-wetting walls under a direct current (DC) electric field. The main channel and the branches on the junction have the same width (*w* = 100 μm). The length of the main channel is 8*w* (*L* = 0.8 mm), and the horizontal ones are both 5*w* (*z* = 1.1* mm*). As shown in Fig. 1, the droplet initially has a rectangular shape with the initial length of *L*_0_, density *ρ*_d_, viscosity *μ*_d_, and relative permittivity of *ε*_*d*_ is moved by the continuous phase with density *ρ*_c_, viscosity *μ*_c_, and relative permittivity *ε*_*c*_. The rear of droplet is placed *L*_0_/*w* = 0.3 from the inlet. A parabolic velocity profile is imposed on the inlet:

1 $$\left\{ {\begin{array}{*{20}l} {u = 0} \hfill \\ {v = - U_{c} \left( {1 - \left( {\frac{{2 \left( {x - x_{0} } \right)}}{w}} \right)^{2} } \right)} \hfill \\ \end{array} .} \right.$$

where *u* (m/s) and *v* (m/s) are the velocity components along the *x* and y directions, respectively, and *y*_*0*_ is the *y-*coordinate of the centerline of the vertical channel. Furthermore, a non-slip boundary condition is implemented for all the solid walls (*u* = *v* = 0). Also, a uniform relative pressure is set to a 0 Pa gauge pressure condition. A steady and uniform electric field (*E*) is generated along the *y*-direction as 0 and *V*_*0*_ are exerted to the lower wall and the upper wall of the left branch, respectively. Additionally, a zero-charge condition is applied for other boundaries.

The physical properties of fluids are presented in Table 1.

**Table 1:** Physical properties of droplet and the surrounding fluid^14^.

	Density (kg/m^3^)	Viscosity (mPa s)	Relative permittivity	Surface tension (N/m)
Disperse fluid	1000	1	78.5 *ε*_0_	0.0033
Continuous fluid	930	10	2.8 *ε*_0_	

The following non-dimensional numbers are introduced that describe the problem;

$${\text{Ca}} = \frac{{\mu_{c} U_{c} }}{\gamma } \cdot {\text{L}}^{*} = \frac{{L_{0} }}{w} \cdot \rho^{*} = \frac{{\rho_{d} }}{{\rho_{c} }} \cdot \mu^{*} = \frac{{\mu_{d} }}{{\mu_{c} }} \cdot {\text{Ca}}_{{\text{e}}} = \frac{{\varepsilon_{0} \varepsilon_{c} w E^{2} }}{\gamma } {\text{and}}\quad \varepsilon^{*} = \frac{{\varepsilon_{d} }}{{\varepsilon_{c} }}$$

where *ρ* is density, *μ* viscosity, *U*_*c*_ is the inlet velocity of continuous phases, *L*_*0*_ is initial length of the droplet, relative permittivity is *ε*, *E* representing the uniform electric field strength, and interfacial tension between phases is shown by *γ*. The subscripts *c* and *d* represent continuous and dispersed phases, respectively. The density ratio (ρ^*^), viscosity ratio (μ^*^), and permittivity ratio (ε^*^) are kept constant for all the investigated cases. Electrical Capillary number (Ca_e_) and permittivity ratio (ε^*^) are two factors used to represent the effect of the electric field on the process of droplet motion. The electrical Capillary number represents the ratio between the electric force and the interfacial tension force. Permittivity ratio describes the fluid response to the applied electric field. In this simulation, both phases are assumed incompressible and dielectric, and the permittivity of the continuous phase is assumed to be equal to the permittivity of the vacuum (*ε*_0_).

## Mathematical models

To investigate the dynamics of the droplet deformation, the Navier–Stokes equation should be solved numerically. We use the level-set method to capture the interface of the two immiscible phases. Besides, the interfacial force and the electrical force are added to the Navier–Stokes equation as source terms;

2 $$\nabla \cdot {\varvec{u}} = 0.$$

and

3 $$\rho \frac{{\partial {\varvec{u}}}}{{\partial {\text{t}}}} + \rho \left( {{\varvec{u}} \cdot \nabla } \right){\varvec{u}} = - \nabla p + \nabla \cdot \left[ {\mu \left( {\nabla { }{\varvec{u}} + \left( {\nabla {\varvec{u}}} \right)^{T} } \right)} \right] + {\varvec{F}}_{e} + {\varvec{F}}_{\gamma } .$$

where *p*, *ρ*, ***u***, ***F***_***e***_ and ***F***_*γ*_ represent the fluid pressure, the fluid density, the velocity vector, the electric force and the interfacial force of two immiscible fluids, respectively. Due to the small size of the microchannel and small density ratio, gravity is neglected.

By solving the transport equation of the Level-set as

4 $$\frac{\partial \Phi }{{\partial {\text{t}}}} + \nabla \cdot \left( {{\varvec{u}} \Phi } \right) = \lambda \nabla \cdot \left(\epsilon {\nabla \Phi - \Phi \left( {1 - \Phi } \right)\frac{\nabla \Phi }{{\left| {\nabla \Phi } \right|}}} \right).$$

the interface will be captured. Here, Φ*,* λ and $\epsilon$ are level-set function, reinitialization parameter and interface thickness parameter, respectively.

The level-set function (Φ) is defined as:

5 $$\Phi = \left\{ {\begin{array}{*{20}l} {1.} \hfill & {\quad {\text{dispersed phase}}} \hfill \\ {0 < \Phi < 1.} \hfill & {\quad {\text{interface}}} \hfill \\ {0.} \hfill & {\quad {\text{continuous phase}}} \hfill \\ \end{array} } \right.$$

The surface tension force on the interface can be calculated as follows:

6 $${\varvec{F}}_{\gamma } = \gamma \kappa \delta {\varvec{n}}.$$

where *γ*, ***κ*** and ***n*** denote the surface tension coefficient, the curvature of the interface, and the normal direction with respect to the droplet surface, respectively. *δ* is a Dirac Delta function.

The electric force is calculated by solving the distribution of the electric field based on the location and shape of the droplet. By neglecting the magnetic induction effect due to the small dynamic currents, the electric field can be viewed as irrational^32^. The charge conservation can be written as:

7 $${ }\frac{{{\text{D}}q_{{\varvec{v}}} }}{{{\text{Dt}}}} + \nabla \cdot \left( {\sigma {\varvec{E}}} \right) = 0,$$

where *q*_v_ is the volume density of local free charges, *σ* represents the conductivity of the fluid. From Maxwell's equations, the electrical relaxation time of the droplet is given by $\tau_{c} = \frac{{\varepsilon_{w} }}{{\sigma_{w} }}$. For our simulations, *ε*_*w*_ = 80*ε*_0_, and *σ*_*w*_ = 5.0 × 10^−4^ S/m, yielding $\tau_{c} \approx 1.4 \times 10^{ - 6} \,{\text{s}}$. The charge accumulation at the interface happens much faster compared to the time scale of fluid motion^33^. Hence, the first term of Eq. (7) can be ignored and it can be simplified as:

8 $$\nabla \cdot \left( {\sigma {\varvec{E}}} \right) = 0.$$

The electric field intensity can be calculated in terms of electric potential by ${\varvec{E}} = - \nabla V$. Thus Eq. (8) is further written as:

9 $$\nabla \cdot \left( {\sigma \nabla V} \right) = 0.$$

The distribution of electric potential is obtained by solving Eq. (9). The electric displacement (***D*** = *ε*_0_*ε****E***) are also calculated accordingly. The divergence of the Maxwell stress tensor can determine the electric force as

10 $${\varvec{M}} = {\varvec{ED}} - \frac{1}{2}\left( {{\varvec{E}} \cdot {\varvec{D}}} \right){\varvec{I}}.$$

11 $${\varvec{F}}_{e} = \nabla \cdot {\varvec{M}} = q_{{\varvec{v}}} {\varvec{E}} - \frac{1}{2}E^{2} \nabla \varepsilon_{0} \varepsilon .$$

With the condition of no existence of free change (*q*_*v*_ = 0) and incompressible fluid, Eq. (11) can be simplified as:

12 $${\varvec{F}}_{e} = - \frac{1}{2}E^{2} \varepsilon_{0} \nabla \varepsilon .$$

## Numerical methods

**Figure 2:**
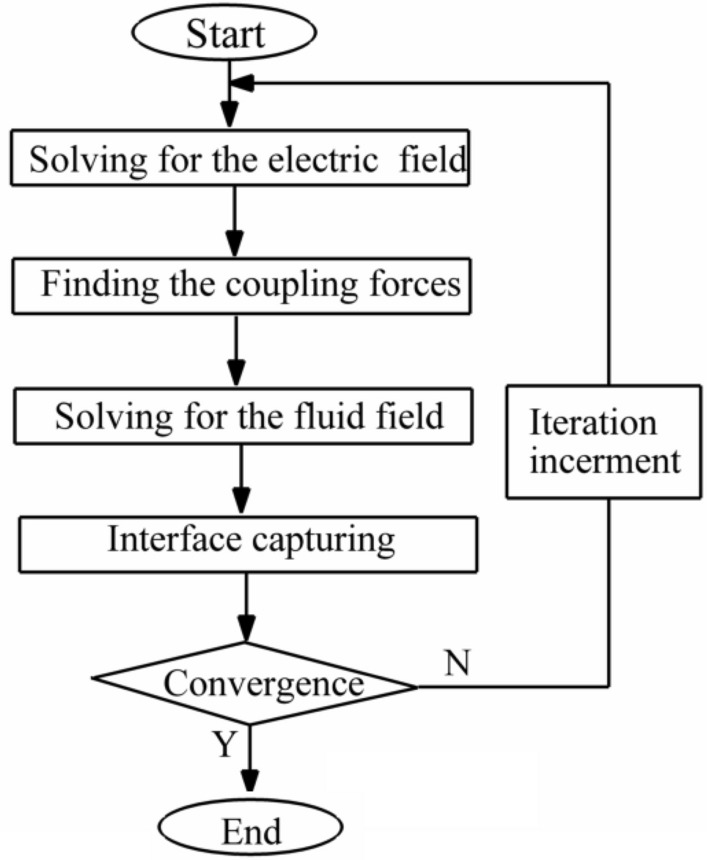
A flowchart of the algorithm for the numerical method.

In recent years, the computational fluid dynamics (CFD) method has been implemented in industries to model real engineering problems^34–51^. To this approach, the COMSOL Multi-physics software is applied to simulate the motion of droplets under the electric field. The laminar Two-Phase level-set method in the fluid dynamics module is used to simulate the motion of the droplet in a symmetric micro-sized T-junction under an electric field. The Poisson equation is solved in Electrostatic Interface in AC/DC Module to compute the electric field. A flowchart of the algorithm for the current simulation is displayed in Fig. 2.

## Results and discussion

### Model validation

To validate current results, two different cases are considered. First, the droplet motion in the microchannel is simulated to verify the flow field. Second, the deformation of a static droplet under the electric field is also examined to validate the application of the electric field.

#### Droplet breakup in a symmetric T-junction microchannel

**Figure 3:**
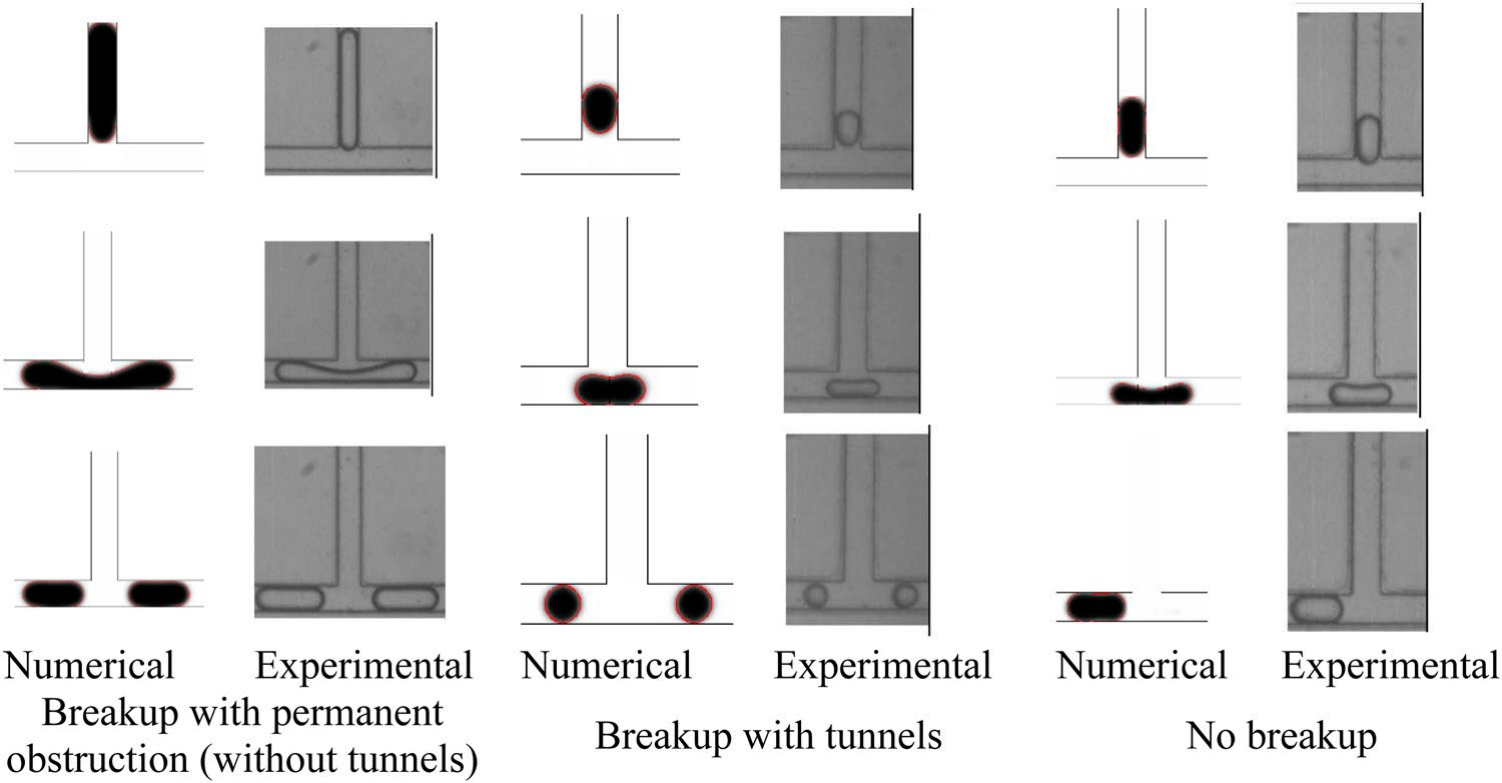
Qualitative comparison of different patterns predicted by simulation with that observed in experiments (Jullien et al.^22^).

Figure 3 displays a qualitative comparison between the experiments conducted by Jullien et al.^22^ and the numerical results. They classified droplet motion processes in symmetric T-junctions microchannel into three different patterns as no breakup, breakup with tunnels, and break up with permanent obstruction patterns (without tunnels). The comparison reveals that the results have good agreement with experimental results in terms of the flow patterns.

**Figure 4:**
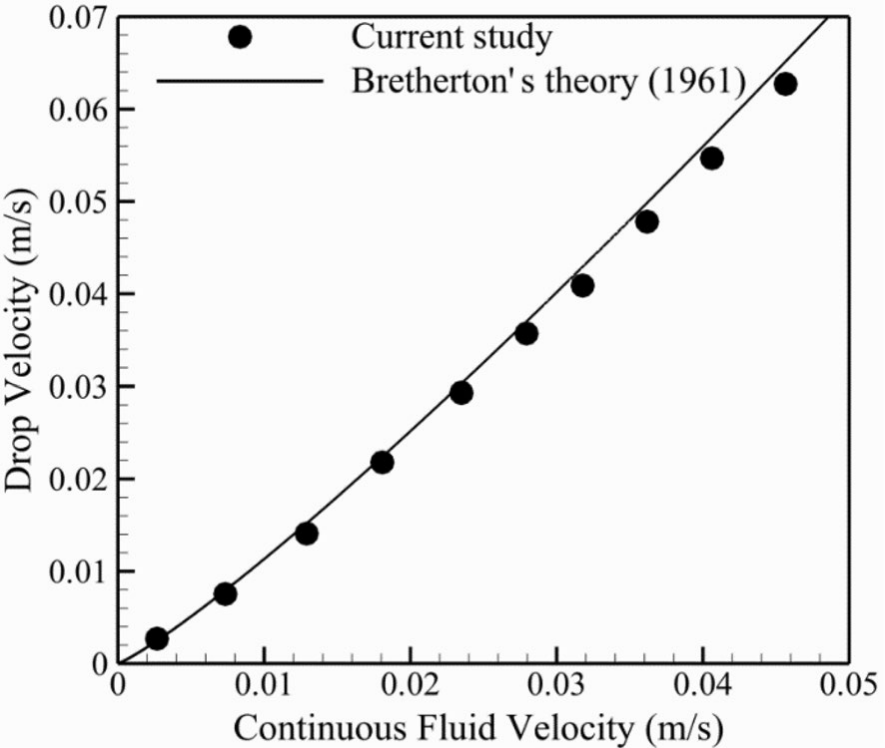
The comparison between the present result and Bretherton^52^ analytical relation.

Figure 4 presents the comparison between the present result and Bretherton^52^ analytical relation. Assuming no contact between the wall and the disperse phase and small Reynolds number flow, Bretherton^52^ suggested an analytical correlation for the velocity of the disperse phase in a slender tube (*U*) with respect to the viscosity of continuous phase (*μ*_*c*_), surface tension (*γ*), and the average velocity of the fluid flow ($\overline{U}$), as follow:

13 $$U = \overline{U}\left( {1 + 1.29\left( {\frac{{\mu_{c} u_{c} }}{\gamma }} \right)^{\frac{2}{3}} } \right).$$

As can be seen, present results match excellently with Eq. (13).

#### Equilibrium shape of the droplet under an electric field

**Figure 5:**
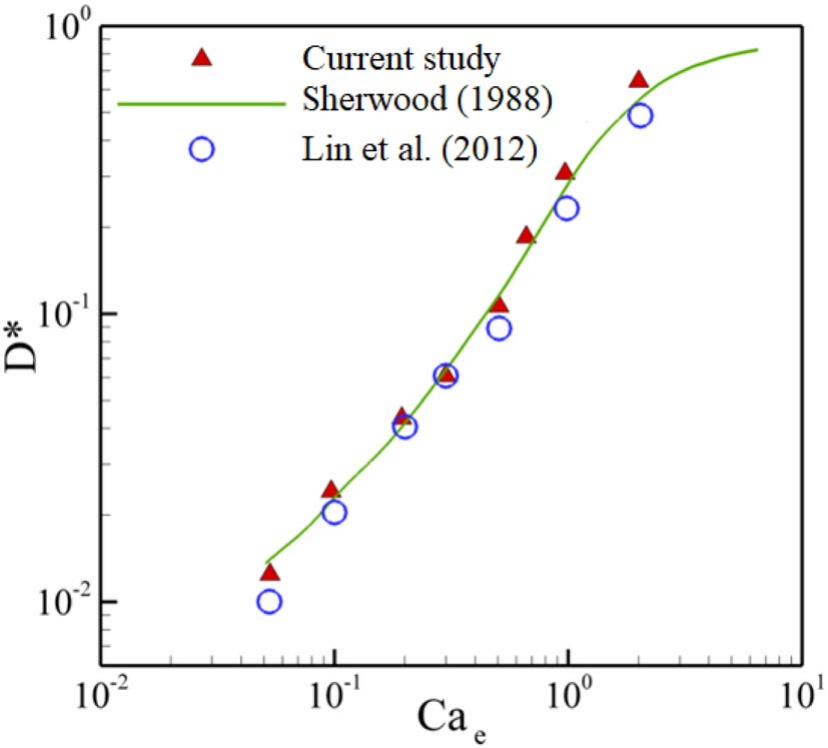
A comparison between deformation parameter and electric capillary number by present method with Sherwood^33^ and Lin et al.^53^.

To validate the coupling of the electric field and the flow field, a simulation is performed to analyze the equilibrium shape of a droplet in a non-electric fluid under a uniform electric field in the *y*-direction. Figure 5 shows the relationship between deformation parameter D^*^ and electrical Capillary number. D^*^ is defined as $\frac{a - b}{{a + b}}$ in which *a* and *b* denote the major and minor axes of the deformed droplet, respectively. The simulation is compared with the theoretical prediction of Sherwood^33^ and Lin et al.^53^. As can be seen in Fig. 5, the current results are in good agreement with analytical and numerical results.

From Figs. 3, 4, and 5, it can be concluded that the current model is an appropriate method to simulate the droplet behaviors in symmetric T-junction microchannel under uniform electric fields.

### Grid independence study

**Figure 6:**
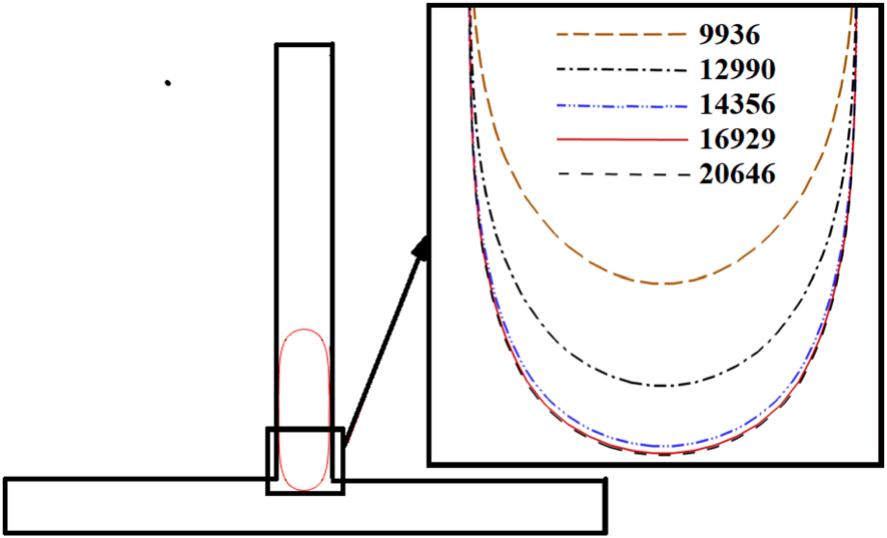
Effect of mesh size on the interface of the droplet in a symmetric T-junction in a moment that droplet achieves in the junction.

To ensure that the current study results are independent of grid sizes, the interface of the droplet is simulated in a symmetric T-junction in the moment that the mother droplet reaches the junction, as shown in Fig. 6. Five triangular elements containing 9936, 12,990, 14,356, 16,929, and 20,646 rectangular elements are tested. It is observed that the change of the droplet interface is negligible for the grid with more than 16,929 elements. Consequently, the grid with 16,929 rectangular elements is considered for present simulations for further studies.

### Droplet motion under the electric field

In this section, we investigate the motion behavior of the droplet when it passes through the T-junction microchannel. The electric field is applied to the left branch of the T-junction, and this results in an asymmetric force field that can cause droplet splitting. Different splitting regimes were observed which is due to the dimensionless length of the mother droplet, L^*^, and the electrical capillary number, Ca_e_. In the following, we explain how the presence of the electric field alters the microdroplet breakup and results in the formation of different breakup regimes.

**Figure 7:**
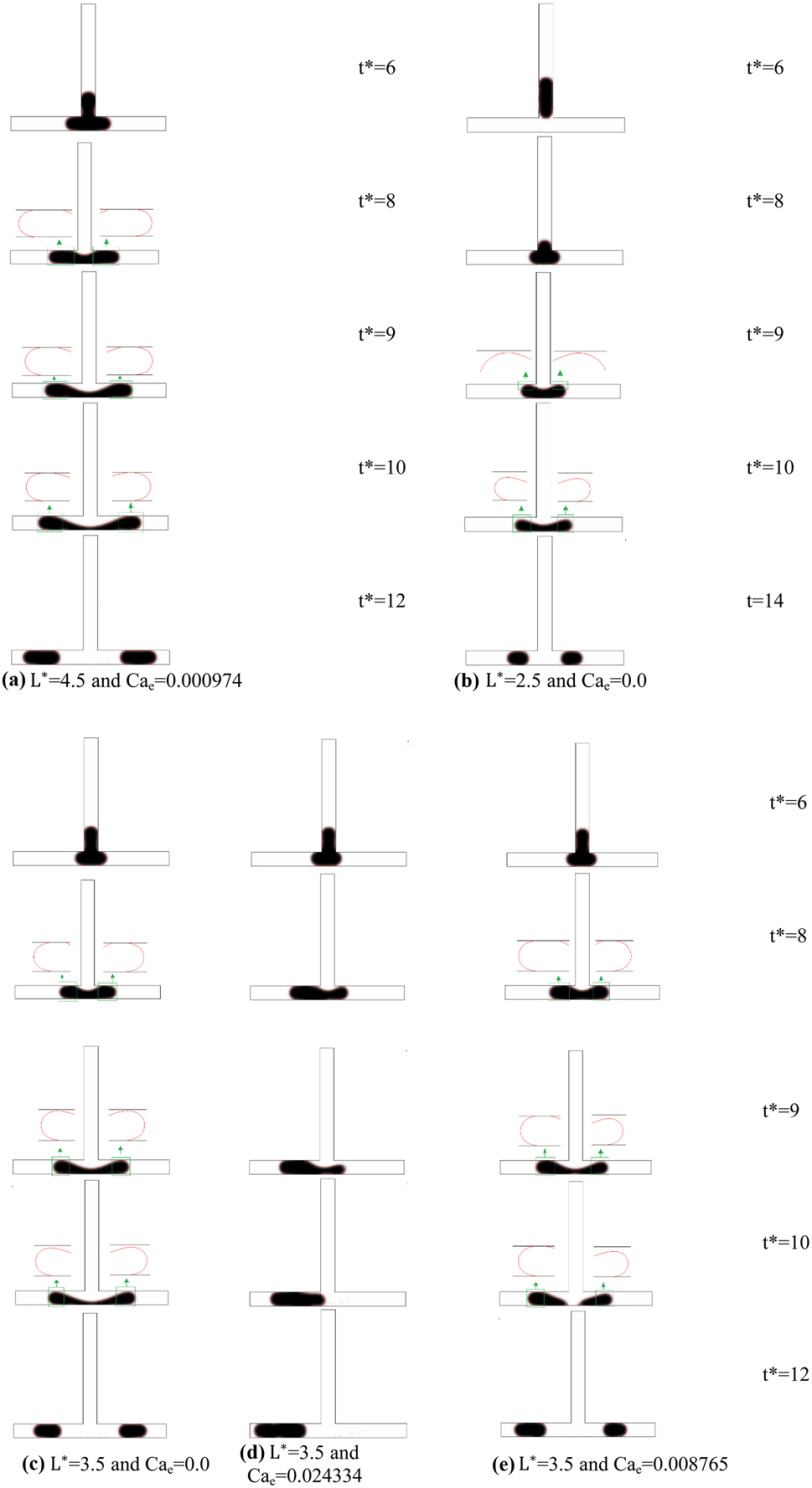
Time evolution of droplet motion at the symmetric T-junction for ε^*^ = 36.4, Ca = 0.02684: L^*^: (**a**) splitting with permanent obstruction (SPO), (**b**) splitting with temporary obstruction (STO), (**c**) splitting with tunnel (SWT), (**d**) asymmetric splitting mode (ASM), and (**e**) non-splitting droplet (NS).

Figure 7a–e present the time evolution of the microdroplets dynamic. Five different splitting regimes were observed based on the droplet breakage pattern and the blockage of the channel branches. The first regime shown in Fig. 7a, is due to the permanent obstruction of the channel. When the droplet reaches the bottom of the channel at t^*^ = 6, the droplet starts to stretch, and the layer connecting the two bulbs of the droplet will become thinner. With further development in time, the layer will become thinner. However, both sides block the channel branches, and this creates a pressure droplet gradient between the tip of the droplet bulbs and the center. This pressure will increase until the connecting layer decreases and the breakage happens. Since the main reason for this splitting is caused by the permanent obstruction of the channel branches, this regime is called Splitting with Permanent Obstruction (SPO). The second regime that we observe in the simulation result has a similar pattern to the SPO. However, by decreasing the size of the mother droplet and the electric force, the bulbs of the droplet on the junction cannot fill the whole channel, and therefore a tunnel will be created. These tunnels, which are shown in Fig. 7b at t^*^ = 10, lead to a lower pressure gradient in comparison to the SPO. But, this pressure is still enough for the droplet splitting, and we call this regime Splitting with Tunnel (SWT). Figure 7c presents the time evolution of a breakage regime, which happens in the length scale between the two above-mentioned regimes. In this case, the droplet starts to occupy the whole channel in the junction. However, neither the size of the droplet nor the electric force is enough to keep the connection of the droplet to the sidewalls. Therefore, a temporary blockage happens, and by increasing the pressure in the time, tunnels will be created. This regime is so-called Splitting with Temporary Obstruction (STO). At a high electrical capillary number, the droplet does not split up just like the non-splitting (NS) regime in the symmetric T-junction and moves entirely into the left side of the channel, as depicted in Fig. 7d. In the presence of strong electrical force, the whole droplet is dragged towards the left side of the channel, and no breakage happens. It is worth mentioning that the unbroken droplet always flows towards the left channel due to a higher electric field, unlike the symmetric T-junction that randomly flows into either left or right of the outlet branch. Hence, we call this regime, the sorting regime (SR). Due to the one-sided electric field in the symmetric T-junction, a new flow regime is revealed, which we introduce as Hybrid Asymmetric Splitting Mode (HASM). According to Fig. 7e, in this case, when the microdroplet enters the symmetric T-junction and blocks the channel, the continuous fluid starts applying pressure to the microdroplet in the junction. The applied electric force on the left side of the channel pulls the droplet. Now, if the force intensity is not strong enough to drag the two tips towards itself, tunnels will be created, and the continuous fluid flow through the tunnels would form an STO regime. If the intensity of the electric force is high enough to keep the channel branches blocked, only on the right side of the channel, the tunnel will be created, while no flow between sidewalls and the droplet is observed for the left branch. This flow regime creates Hybrid Asymmetric Splitting Mode (HASM).

**Figure 8:**
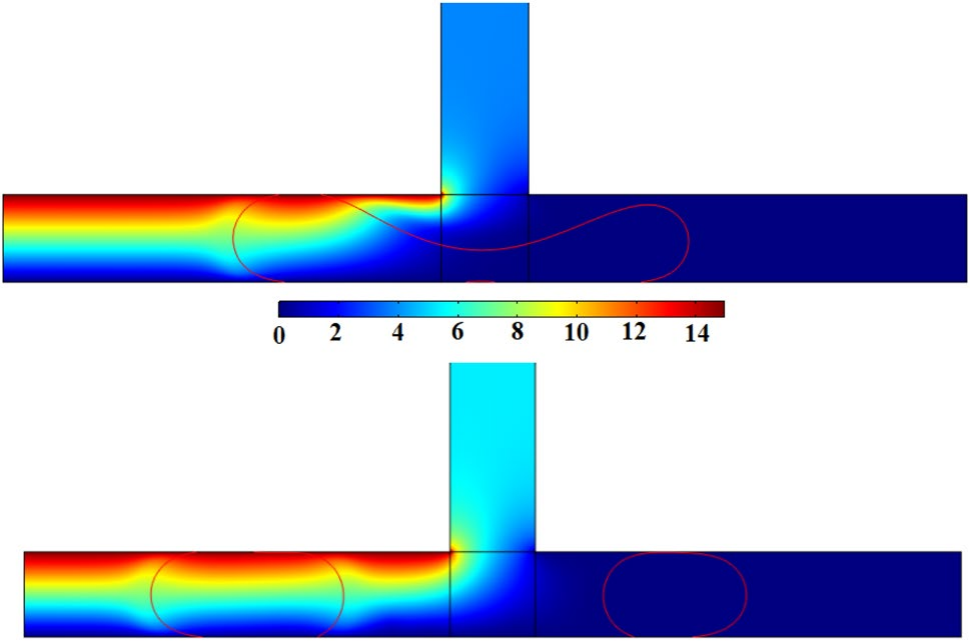
The contour of the electric potential for L^*^ = 3.5 ε^*^ = 36.4, Ca = 0.02684 and Ca_e_ = 0.008765 at *V* = 15 Volt : (a) t^*^ = 9 and (b) t^*^ = 11.

**Figure 9:**
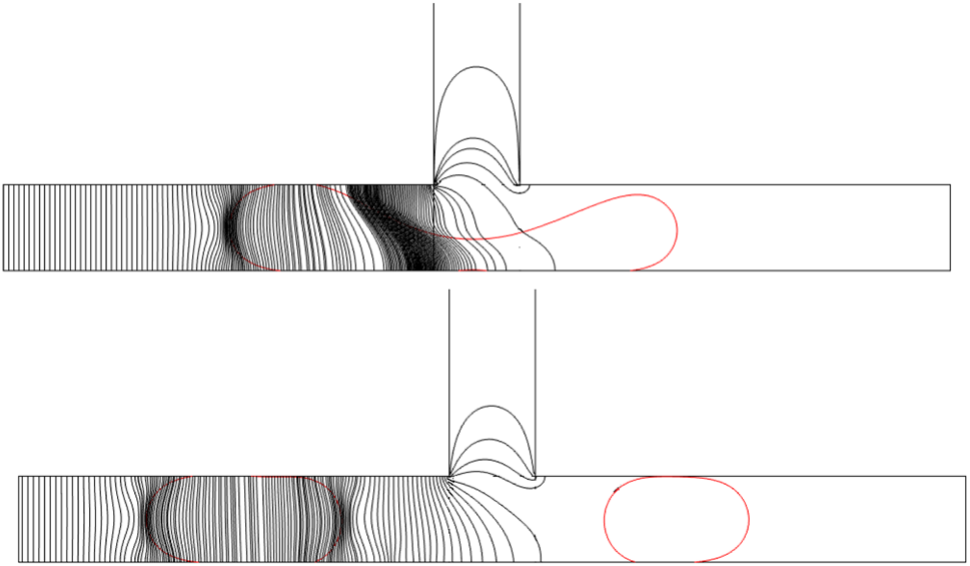
Electric field lines for L^*^ = 3.5 ε^*^ = 36.4, Ca = 0.02684 and Ca_e_ = 0.008765: (a) t^*^ = 9 and (b) t^*^ = 11.

**Figure 10:**
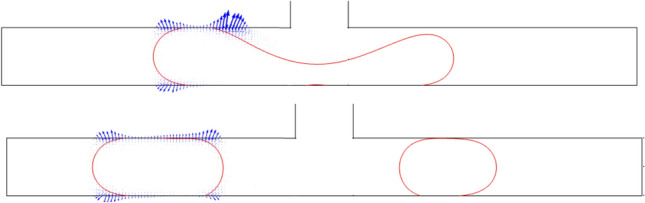
Electric field force distributions on the droplet for L^*^ = 3.5 ε^*^ = 36.4, Ca = 0.02684 and Ca_e_ = 0.008765: (**a**) t^*^ = 9 and (**b**) t^*^ = 11.

To further investigate these phenomena, Figs. 8 and 9 present the electric potential and electric field lines for L^*^ = 2.5 ε^*^ = 36.4, Ca = 0.0134 and Ca_e_ = 0.0351, respectively. When the daughter droplet enters the left channel, the local electric potential and electric field change due to the change in the dielectric constant of the medium. Inside the droplet, however, these field lines are constantly distributed. Therefore, a non-uniform force field will be formed around the droplet. Since the electric field is stronger in a medium with higher permittivity, a stronger electric field is created inside the droplet leading to an electric gradient at the surface of the droplet. This leads to a force at the droplet surface pointing from the inside of the droplet to the outside (as depicted in Fig. 10)^54^. If the force is strong enough, this leads to an obstruction, which is explained above. On the other side of the junction, in the absence of the electric field, there is no additional force to pull the droplet towards the side walls. Therefore, a tunnel may be formed there, which allows the continuous phase to pass through. This will additionally create a pressure gradient that pushes the mother droplet to the left, as is shown in Fig. 10. As a result, a stronger squeezing force acts on the shifted neck of the mother droplet, which creates an asymmetric droplet breakup.

**Figure 11:**
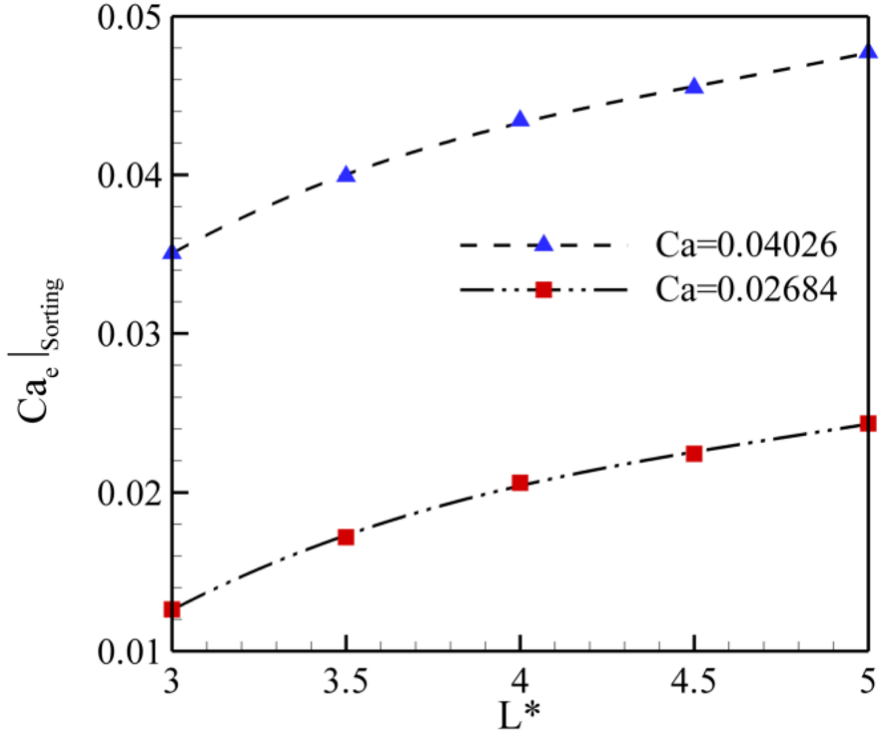
Variation of the sorting electrical capillary number versus to dimensionless droplet length (L^*^) for Ca = 0.02684 and 0.04026.

As mentioned in Fig. 7d, the mother droplet does not break and is sorted on the left side of the branch after a certain electric capillary number (hereinafter it is called the sorting electric capillary number and denoted by the symbol $\left. {Ca_{e} } \right|_{Sorting}$). Figure 11 displays the variation of the sorting electrical capillary number versus to dimensionless droplet length (L^*^) for Ca = 0.02684 and 0.04026. As can be seen, $\left. {Ca_{e} } \right|_{Sorting}$ increases with the increment of L^*^. It is worth mentioning that this increment is more highlighted for ca = 0.02684. Achieved results indicates $\left. {Ca_{e} } \right|_{Sorting}$ increases about 93% when the L^*^ is raised from L^*^ = 3.0 to L^*^ = 5.0 = 0.0268. However, raising Capillary number from Ca = 0.02684 to Ca = 0.04026 (about 50%) just increase $\left. {Ca_{e} } \right|_{Sorting}$ about 36%. Also, a correlation the sorting electric capillary number as a function of L^*^ could be attained by the fitting numerical data as $\left. {Ca_{e} } \right|_{Sorting} = a L^{*2} + bL^{*} + c{ }$ where *a*, *b, and c* are reported in Table 2.

**Table 2:** *a*, *b*, and *c* values for correlation the sorting electric capillary number as a function of L^*^ ($\left. {Ca_{e} } \right|_{Sorting} = a L^{*2} + bL^{*} + c{ }$).

	*a*	*b*	*C*	*RMS*
Ca = 0.02684	− 0.002	0.0215	− 0.0341	0.9975
Ca = 0.04026	− 0.0019	0.0216	− 0.0122	0.9969

A major benefit of using the electric field in the T-junctions is to break up precisely a mother droplet into arbitrary sizes. To exploit this benefit efficiently, it is essential to predict the splitting ratio of the droplets (SR^*^). It is defined as:

14 $${\text{SR}}^{*} = \frac{{\text{Area of right daughter droplet}}}{{\text{Area of left daughter droplet}}}.$$

**Figure 12:**
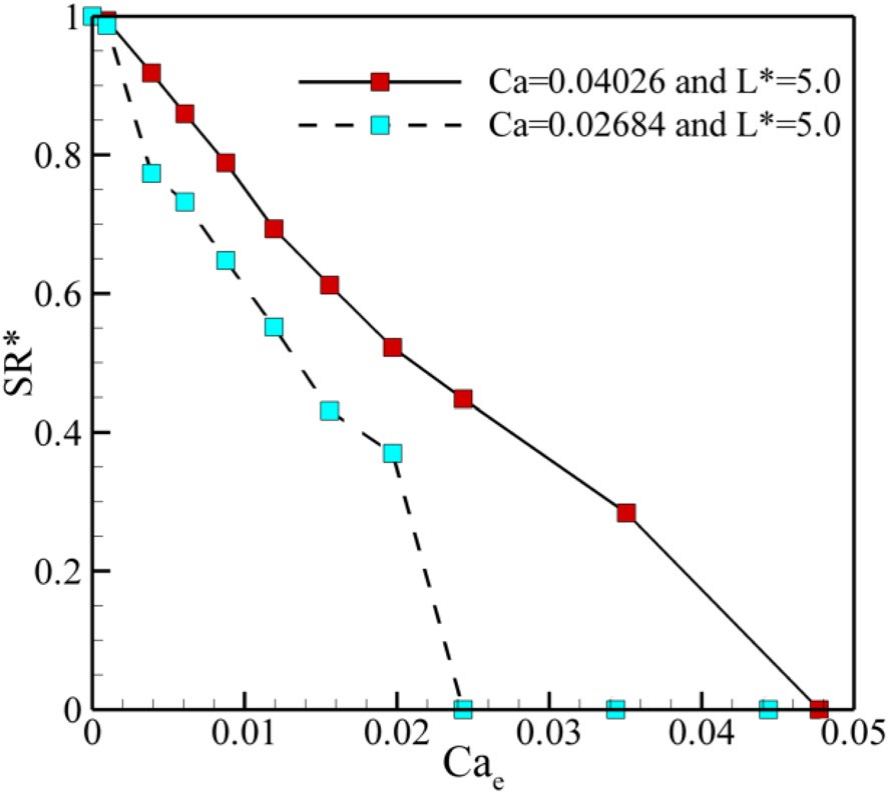
The splitting ratio of the droplets as a function of electric capillary number at L^*^ = 5.0 for Ca = 0.02684 and Ca = 0.04026.

It varies from 0 to 1.0. The splitting ratios of 0 and 1.0 correspond to the sorting and the symmetric breakup, correspondingly. 0 < V^*^ < 1 refers to the mother droplet breaks up asymmetrically. According to the previous studies, splitting of the mother droplet in the absence of an electric field crucially depends on dimensionless droplet length (L^*^) and capillary number (Ca). Another dimensionless that affects is the electric capillary number (Ca_e_). Figure 12 illustrates the effect of Ca_e_ on the splitting ratio at L^*^5.0 for Ca = 0.02684 and Ca = 0.04026, respectively. It is evident from this figure that the splitting ratio decreases with the increase of the Ca_e_ until a critical value. In fact, the electric force relative to the interfacial tension force enhances by the increment of Ca_e_. Hence, more volume of mother droplet is drawn towards the left side of the channel in which mass center of mother droplet is pulled into the left branch, leads to more asymmetric splitting. Further increment of Ca_e_ beyond the critical value results in the whole mother droplet being shifted to the left branch causes to SR^*^ = 0. Furthermore, the droplet with a higher amount of SR^*^ is observed for Ca = 0.04026 compared with Ca = 0.02684 at a constant Ca_e_. Also, a sudden level off up to 0 for SR^*^ happens at lower Ca_e_ as the Ca decreases. It must be mentioned that similar trends are observed for other L^*^, but they are omitted for better view.

## Conclusion

In the current study, the motion of droplet under an asymmetric electric field in the asymmetric T-junction microchannel is investigated numerically. To this approach, COMSOL Multi-physics software based on the Level Set method is adopted. The effects of various factors, namely the non-dimensional droplet length (L^*^), Capillary number (Ca), and electric Capillary number (Ca_e_), are investigated and the following results are drawn:

1. It is observed that the use of an asymmetric electric field directly affects the droplet splitting process and flow patterns in symmetric T-junctions microchannel and produces droplets with unequal size.
2. Two novel patterns of droplets named Hybrid Asymmetric Splitting Mode (HASM) and sorting patterns may happen when the electric field imposes asymmetrically.
3. There is a critical electric capillary number above (the sorting electric capillary number) that the non-splitting (NS) regime occurs for each dimensionless length of mother droplet (L^*^) and Capillary number resulting in the splitting ratio of the droplets (SR^*^) 0.
4. At a constant Ca_e_, the droplet with a higher amount of SR^*^ is observed for Ca = 0.04026 compared with Ca = 0.02684. Also, a sudden level off up to 0 for SR^*^ happens at lower Ca_e_ as the Ca decreases.
5. The current results indicate that the proposed novel method is a suitable approach to adjust actively the size of droplets.
